# Broad protection and respiratory immunity of dual mRNA vaccination against SARS-CoV-2 variants

**DOI:** 10.1038/s41541-024-00957-2

**Published:** 2024-09-04

**Authors:** Renee L. Hajnik, Jessica A. Plante, Srinivasa Reddy Bonam, Grace H. Rafael, Yuejin Liang, Nicholas C. Hazell, Jordyn Walker, Rachel A. Reyna, David H. Walker, Mohamad-Gabriel Alameh, Drew Weissman, Scott C. Weaver, Kenneth S. Plante, Haitao Hu

**Affiliations:** 1 Department of Microbiology and Immunology, University of Texas Medical Branch, Galveston, TX USA; 2 Department of Pathology, University of Texas Medical Branch, Galveston, TX USA; 3 Institute for Human Infections and Immunity, University of Texas Medical Branch, Galveston, TX USA; 4 World Reference Center for Emerging Viruses and Arboviruses, University of Texas Medical Branch, Galveston, TX USA; 5 Sealy Institute for Vaccine Sciences, University of Texas Medical Branch, Galveston, TX USA; 6 Department of Medicine, University of Pennsylvania Perelman School of Medicine, Philadelphia, PA USA; 7 Penn Institute for RNA Innovation, University of Pennsylvania Perelman School of Medicine, Philadelphia, PA USA

**Keywords:** RNA vaccines, Vaccines

## Abstract

While first-generation, spike (S)-based COVID-19 vaccines were effective against early SARS-CoV-2 strains, the rapid evolution of novel Omicron subvariants have substantially reduced vaccine efficacy. As such, broadly protective vaccines against SARS-CoV-2 are needed to prevent future viral emergence. In addition, it remains less clear whether peripheral immunization, especially with mRNA vaccines, elicits effective respiratory immunity. Our group has developed a nucleoside-modified mRNA vaccine expressing the nucleocapsid (N) protein of the ancestral SARS-CoV-2 virus and has tested its use in combination with the S-based mRNA vaccine (mRNA-S). In this study, we examined efficacy of mRNA-N alone or in combination with mRNA-S (mRNA-S+N) against more immune evasive Omicron variants in hamsters. Our data show that mRNA-N alone induces a modest but significant protection against BA.5 and that dual mRNA-S+N vaccination confers complete protection against both BA.5 and BQ.1, preventing detection of virus in the hamster lungs. Analysis of respiratory immune response in mice shows that intramuscular mRNA-S+N immunization effectively induces respiratory S- and N-specific T cell responses in the lungs and in bronchoalveolar lavage (BAL), as well as antigen-specific binding IgG in BAL. Together, our data further support mRNA-S+N as a potential pan-COVID-19 vaccine for broad protection against current and emerging SARS-CoV-2 variants.

## Introduction

Since 2020, tremendous efforts have been devoted to the development of vaccines for severe acute respiratory syndrome coronavirus 2 (SARS-CoV-2), the causative agent of COVID-19. Two vaccines based on the mRNA-lipid nanoparticle (LNP) platform were rapidly developed and clinically approved. These first-generation mRNA vaccines targeted the viral spike (S) protein and showed high clinical efficacy against the early strains^1,2^. However, a number of immune-escaping SARS-CoV-2 variants have emerged since 2020, including Alpha, Beta, Gamma, Delta, and Omicron variants, largely due to mutations in the viral S protein^3,4^. A number of new Omicron subvariants have also emerged, including BA.2, BA.3, BA.4, BA.5, BQ.1, and others^5^. Extensive pre-clinical and clinical studies have shown that the first-generation vaccines had reduced efficacy against SARS-CoV-2 variants, especially the Omicron strains^6,7^. The emergence of variants has posed continuing challenges to host immunity induced by the monovalent or bivalent S-based vaccines. Thus, development of a pan-SARS-CoV-2 vaccine with broad protection against current and future variants is needed.

A broadly protective SARS-CoV-2 vaccine likely requires targeting the conserved viral epitopes, in addition to the S protein. The viral nucleocapsid (N) protein is relatively conserved across SARS-CoV-2 variants^8^ and is a main target of host immunity in SARS-CoV-2-infected individuals^9,10^. In our previous study, we generated a nucleoside-modified mRNA vaccine that expresses the SARS-CoV-2 N protein (mRNA-N) and showed that vaccination with dual mRNA-S+N, both based on the prototypical viral sequence, conferred strong protection against both Delta and Omicron BA.1 variants in animal models^11^. However, the breadth of protection by this mRNA vaccine approach against newer, more immune evasive SARS-CoV-2 variants^12–14^ remains to be determined. In addition, clinically approved mRNA vaccines are administered via the intramuscular (IM) route. Our previous study reported induction of strong systemic immunity by IM mRNA vaccination^11^. Whether peripheral mRNA vaccination elicits effective antigen-specific respiratory immunity is less clear^15–18^. Addressing these questions will not only facilitate the development of a pan-SARS-CoV-2 vaccine, but also have implications for vaccine development against other respiratory infections.

In this study, we further evaluated efficacy of mRNA-N alone or in combination with mRNA-S (mRNA-S+N) against two newer SARS-CoV-2 Omicron variants (BA.5 and BQ.1) in Syrian hamster models. Our results show that mRNA-N alone induces significant albeit incomplete protection against BA.5 in hamsters. Importantly, dual mRNA-S+N vaccination confers complete protection against both BA.5 and BQ.1, preventing detectable viral loads in the hamster lungs. Serum antibody analysis indicates that the complete protection by mRNA-S+N is independent of neutralizing antibodies against these two variants, further supporting a role of T cell immunity in the vaccine-induced protection. In the C57BL/6 mouse model, we show that IM mRNA-S+N vaccination effectively elicits respiratory S- and N-specific T cell responses in the lungs and bronchoalveolar lavage (BAL). The data provides immunological insights for the robust viral control of immune-escaping SARS-CoV-2 variants by peripheral mRNA-S+N vaccination even in the absence of detectable neutralizing antibodies.

## Results

### mRNA-N alone induces incomplete but significant protection against SARS-CoV-2 Omicron BA.5 variant in hamsters

The coronavirus N protein exhibits higher sequence conservation when compared to the S protein^19,20^. Our previous study reported the development of a nucleoside-modified mRNA vaccine expressing ancestral SARS-CoV-2 N (mRNA-N)^11^. Prior to investigating the extent of protection conferred by mRNA-N against newer variants, we conducted an analysis of the sequence diversity of the N protein among various SARS-CoV-2 strains (BA.1, BA.5, and BQ.1 versus Wuhan-Hu-1 strain), compared to the S protein (Table 1). The analysis reveals that compared to Wuhan-Hu-1 strain, the three Omicron variants demonstrate an overall sequence diversity in 53 (out of 1273) residues for the S protein, and 8 (out of 419) residues for the N protein, supporting that N is more conserved among these SARS-CoV-2 strains relative to S (Table 1).

**Table 1:** Residue Diversity Within the Spike and Nucleocapsid Proteins of SARS-CoV-2 strains

		A	BA.1	BA.5	BQ.1
		Wuhan-Hu-1	EHC_C19_2811c	COR-22-063113/2022	MDL 5125
Protein	AA Residue				
Spike	19	T	T	I	I
	24–27	LPPA	LPPA	S	S
	67	A	V	A	A
	69–70	HV	del	del	del
	76	T	T	I	T
	95	T	I	T	T
	142	G	D	D	D
	143–145	VYY	del	VYY	VYY
	211–214	NLVR	IVREPE	NLGR	NLGR
	339	G	D	D	D
	346	R	R	R	T
	371	S	L	F	F
	373	S	P	P	P
	375–376	ST	FT	FA	FA
	405	D	D	N	N
	408	R	R	S	S
	417	K	N	N	N
	440	N	K	K	K
	444	K	K	K	T
	446	G	S	G	G
	452	L	L	R	R
	460	N	N	N	K
	477–478	ST	NK	NK	NK
	484	E	A	A	A
	486	F	F	V	V
	493	Q	R	Q	Q
	496	G	S	G	G
	498	Q	R	R	R
	501	N	Y	Y	Y
	505	Y	H	H	H
	547	T	K	T	T
	614	D	G	G	G
	655	H	Y	Y	Y
	679	N	K	K	K
	681	P	H	H	H
	764	N	K	K	K
	796	D	Y	Y	Y
	856	N	K	N	N
	954	Q	H	H	H
	969	N	K	K	K
	981	L	F	L	L
Nucleocapsid	13	P	L	L	L
	31–33	ERS	del	del	del
	136	E	E	E	D
	203–204	RG	KR	KR	KR
	413	S	S	R	R

Amino acid (AA) diversity within the spike and nucleocapsid proteins. AA residue numbering is based on the Wuhan-Hu-1 strain. Protein sequences are assumed on the basis of nucleotide sequences. The Wuhan-Hu-1 sequence reflects NCBI Reference Sequence NC_045512.2. All other strains reflect the next-generation sequencing results of the viral stocks utilized in this manuscript.

**Fig. 1:**
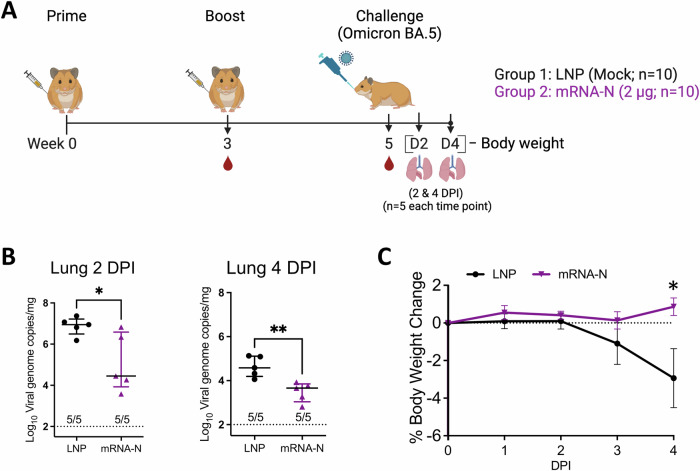
mRNA-N vaccine is efficacious against Omicron variant BA.5 in hamsters. **A** Experimental design and timeline (Created with BioRender.com). Two groups of hamsters (*n* = 10/group) were vaccinated intramuscularly with either empty LNP (mock) or mRNA-N vaccine (2 µg/dose) at weeks 0 and 3. At week 5, hamsters were intranasally challenged with Omicron BA.5 (2 × 10^4^ pfu). **B** Lungs were harvested at 2 and 4 DPI (days post infection; *n* = 5 at each time point) for quantification of viral RNA copies by RT-qPCR. **C** Hamster body weight was monitored from 0 to 4 DPI. In (**B**) symbols represent individual animals, midlines represent the median, error bars represent the interquartile range (IQR), and the dashed lines represent the lower limit of detection (LOD). The number of animals with viral loads above the LOD is noted. Log_10_ normalized data was compared by Mann-Whitney test. In (**C**) symbols represent the mean, error bars represent the standard deviation, and the dashed line highlights 0% weight change. Weight change at individual time point between the two groups was compared by unpaired t-test. *p < 0.05, **p < 0.01.

Next, we assessed the efficacy of mRNA-N against Omicron BA.5 in hamsters, which was a dominant circulating variant in human population and showed substantial neutralization escape^21,22^ when this study was conducted. Two groups of hamsters (*n* = 10 per group) were IM vaccinated with empty LNP (mock) or mRNA-N (2 μg) at weeks 0 and 3, followed by intranasal (IN) challenge with BA.5 (2 × 10^4^ pfu) at week 5 (Fig. 1A). The mRNA vaccine dose was selected based on our recent study^11^. Two (*n* = 5) and four (*n* = 5) days post infection (DPI), hamsters were euthanized and lung tissues were harvested for viral RNA quantification by RT-qPCR (Fig. 1A). Our primary goal was to evaluate the vaccine efficacy based on reductions in viral RNA copies. Since viral replication kinetics for different SARS-CoV-2 strains in animal models varies, lungs are usually harvested at multiple time points after challenge (e.g., 2 and 4 DPI) for quantification of viral loads^11^. Hamster body weights were also monitored daily until the terminal harvest (4 PDI) as a secondary measure of vaccine-induced protection. At 2 DPI, lung viral RNA copies were reduced in the mRNA-N-vaccinated group compared to the mock-vaccinated group (60-fold reduction in mean viral RNA copies; mock versus mRNA-N; *p* < 0.05) (Fig. 1B). At 4 DPI, mRNA-N also induced a significant reduction of lung viral RNA copies (8.3-fold reduction in median copies compared to the mock group; *p* < 0.01; Fig. 1B). Body weight analysis showed that BA.5 infection caused weight reduction in the mock-vaccinated group by ~3% on 4 DPI (Fig. 1C). However, mRNA-N-vaccinated hamsters were protected from weight loss (4 DPI mRNA-N vs. LNP: *p* < 0.05) (Fig. 1C). Together, these data indicated that mRNA-N induced a modest but significant protection against SARS-CoV-2 BA.5. This level of protection is consistent with its efficacy against the Delta strain reported in our previous study^11^.

### Intramuscular immunization with mRNA-N alone induces modest respiratory T cell responses in the lung and bronchoalveolar lavage of mice

Prior results are conflicting regarding whether peripheral mRNA immunization (e.g., IM) elicits effective immunity in the airway, in addition to systemic immunity^15–18^. Our previous study showed induction of systemic T cell responses and circulating antibodies by mRNA-N in BALB/c mice^11^. Here, we investigated respiratory immune responses following IM mRNA-N vaccination in C57BL/6 mice. Use of C57BL/6 mice was in part because SARS-CoV-2 N-epitope specific MHC-I tetramer (H2-Db; N_219-227_: LALLLLDRL)^23^ is available for the identification of N-specific CD8 T cells in the mouse BAL and lung samples. The N_219-227_ is one of the dominant CD8 T cell epitopes within the N protein that was bioinformatically predicted^8^ and then confirmed in humans and mice^24,25^.

**Fig. 2:**
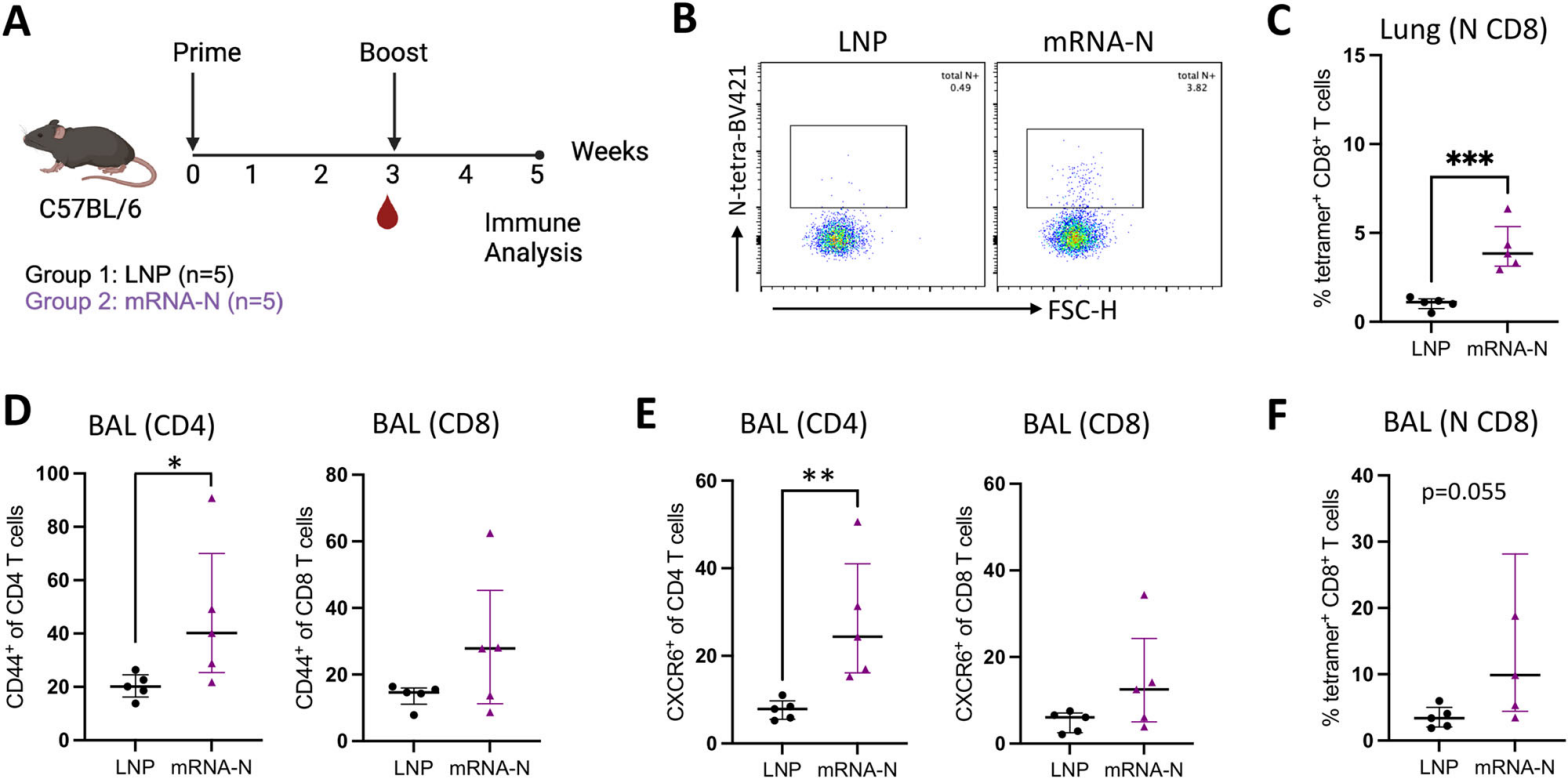
Respiratory T cell response induced by mRNA-N in mice. **A** Mouse experimental design and timeline (Created with BioRender.com). Two groups of C57BL/6 mice (*n* = 5/group) were intramuscularly vaccinated with either empty LNP (mock) or mRNA-N vaccine (1 μg) at weeks 0 and 3. Two weeks after booster dose (week 5), immune responses were analyzed. **B**, **C** Analysis of tetramer positive, N-epitope specific T cells in the lungs by flow cytometry. **B** Representative flow cytometry plots for N-tetramer staining of CD8 T cells from LNP and mRNA-N vaccinated mice. **C** Frequencies of N epitope-specific CD8 T cells in the lungs of LNP and mRNA-N vaccine group. Analysis of total and N epitope-specific T cells in the BAL. Frequencies of activated (CD44^+^) CD4 and CD8 T cells (**D**), CXCR6^+^ CD4 and CD8 T cells (**E**), and N epitope-specific CD8 T cells (**F**) in BAL were examined. Data were presented as median and interquartile range, and were compared by Mann-Whiteney test between the two groups. *p < 0.05, **p < 0.01, ***p < 0.001.

Two groups of C57BL/6 mice (*n* = 5 per group) were IM vaccinated with either empty LNP (mock) or mRNA-N (1 μg) at week 0 (prime) and week 3 (boost). Two weeks after boost vaccination (week 5), lung and BAL samples were collected for analysis of respiratory immune response. Serum and spleen samples were also collected for analysis of systemic immune response for comparison (Fig. 2A). Single cell suspensions prepared from lung and BAL were stained for CD3, CD4, CD8, CD44, CXCR6, and SARS-CoV-2 N-tetramer, to identify activated T cells (based on CD44)^26^, T cells with tissue-homing potential (CXCR6)^27^, and vaccine-elicited, N-specific CD8 T cells (tetramer^+^). Vaccine-induced binding antibodies (IgG and IgA) were also examined in the BAL.

Gating strategy to identify T cell populations and N-tetramer^+^ CD8 T cells in the lungs is shown in Supplementary Fig. 1. Representative flow cytometric plots for N-tetramer^+^ CD8 T cells are shown in Fig. 2B. Compared to mock control, mRNA-N vaccination elicited comparable levels of total CD4 and CD8 T cells in the lung (Supplementary Fig. 2A), and slightly higher levels of activated CD44^+^ (Supplementary Fig. 2B) or CXCR6^+^ (Supplementary Fig. 2C) CD4 and CD8 T cells in the lung. Analysis of N-tetramer straining (Fig. 2B) revealed that compared to mock control, mRNA-N vaccination elicited significant levels of N epitope-specific CD8 T cells in the lungs (*p* < 0.001) (Fig. 2C). Of interest, this N epitope-specific CD8 T cell response was not evident in the spleens after mRNA-N vaccination (Supplementary Fig. 3).

We next evaluated T cell response in the BAL after mRNA-N vaccination. Gating strategy for flow cytometric analysis of BAL cells is shown in Supplementary Fig. 4. Compared to mock control, mRNA-N vaccination elicited higher levels of CD44^+^ CD4 T cells and CD44^+^ CD8 T cells in the BAL, although statistical significance was only achieved for CD4 T cells (*p* < 0.05 for CD4) (Fig. 2D). A similar trend was observed for CXCR6^+^ T cells, with mRNA-N eliciting significantly higher levels of CXCR6^+^ CD4 T cells in the BAL compared to mock control (*p* = 0.01 for CD4) (Fig. 2E). Analysis of N-tetramer^+^ cells revealed that mRNA-N vaccination elicited a trend towards higher levels of N epitope-specific CD8 T cells in the BAL compared to mock control (*p* = 0.055) (Fig. 2F). Analysis of binding antibodies showed that mRNA-N vaccination induced readily detectable N-specific binding IgG in the BAL (Supplementary Fig. 5A). No binding IgA was detected following IM mRNA-N vaccination (Supplementary Fig. 5B), consistent with the previous report^15^. Together, the data indicate that mRNA-N vaccine elicits a modest respiratory immune response including N-specific CD8 T cells in the mouse lungs and BAL.

### mRNA-S+N vaccination induces strong protection against SARS-CoV-2 Omicron BA.5 in hamsters

**Fig. 3:**
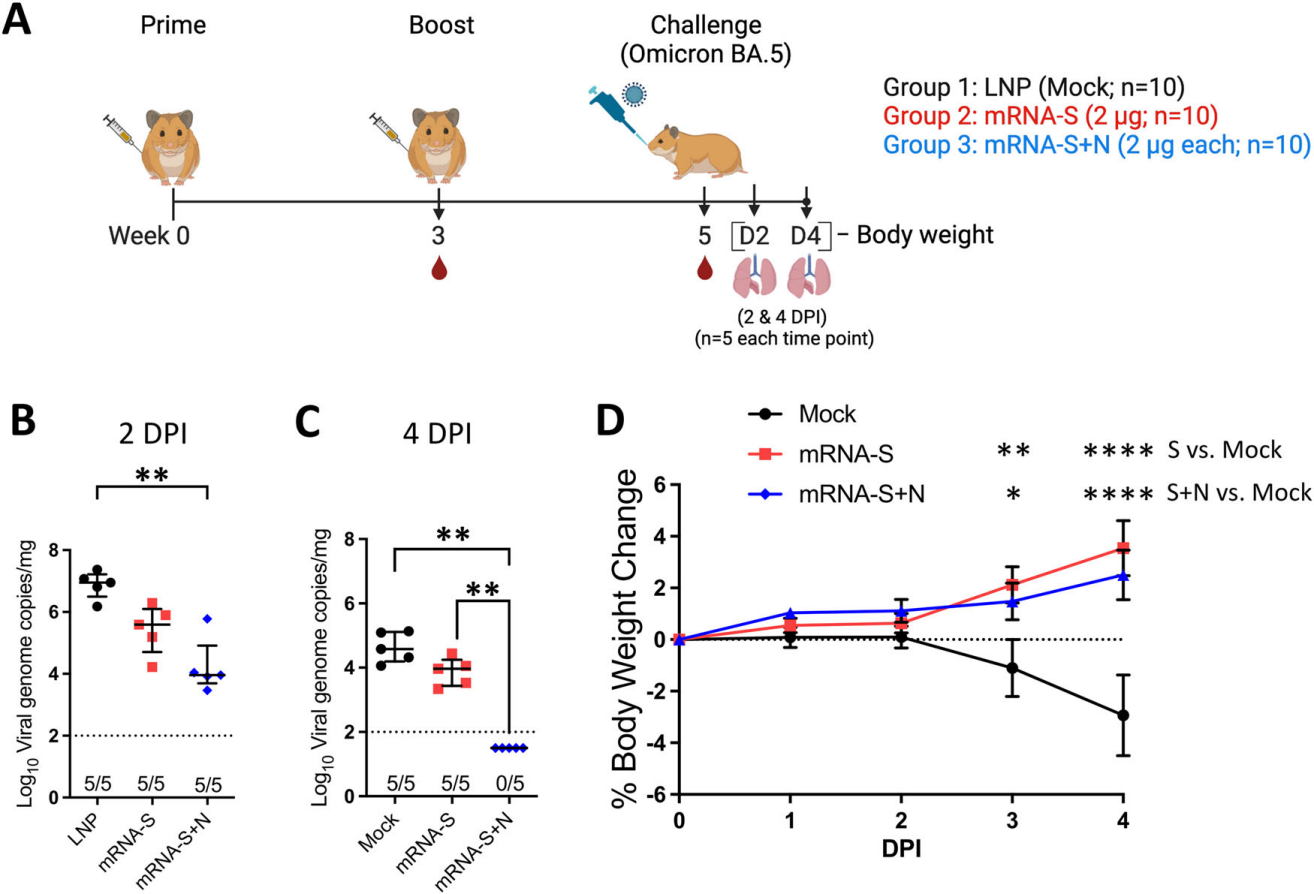
Dual mRNA-S+N vaccination protects hamsters from Omicron BA.5. **A** Hamster experimental design and timeline (Created with BioRender.com). Three groups of hamsters (*n* = 10/group) were vaccinated intramuscularly with empty LNP (mock), mRNA-S (2 μg), or mRNA-S+N (2 μg for each mRNA) at weeks 0 and 3, followed by intranasal challenge with SARS-CoV-2 Omicron BA.5 (2 × 10^4^ pfu) at week 5. Lungs were harvested at 2 (**B**) and 4 (**C**) DPI (*n* = 5 at each time) for quantification of viral RNA copies by RT-qPCR. (**D**) Hamster body weights were monitored from 0 to 4 DPI. In (**B**, **C**), symbols represent individual animals, midlines represent the median, error bars represent the interquartile range, and the dashed line represents the lower limit of detection (LOD). The number of animals with viral loads above the LOD is noted. Data were compared among the three groups by Kruskal-Wallis test. In (**D**), symbols represent the mean, error bars represent the standard deviation, and the dashed line highlights 0% weight change. Weight change was compared by 2-way ANOVA followed by Tukey’s multiple comparisons test (two factors; hamsters’ weight and time). *p < 0.05, **p < 0.01, ****p < 0.0001.

After demonstrating that mRNA-N alone elicited modest protection against BA.5, we next explored whether dual mRNA-S+N vaccination induces stronger protection against this variant than mRNA-S alone. Three groups of hamsters were vaccinated with empty LNP (mock), mRNA-S (2 μg), or mRNA-S+N (2 μg for each mRNA) at weeks 0 and 3, followed by intranasal challenge at week 5. On 2 (*n* = 5) and 4 (*n* = 5) DPI, vaccine-induced protection was analyzed based on viral loads (Fig. 3A). Analysis of viral RNA in the lungs revealed that, compared to mock vaccination, mRNA-S alone induced modest but significant protection, reducing the lung viral RNA copies by 22 and 4 folds at 2 and 4 DPI, respectively (Fig. 3B–C). Relative to mRNA-S alone, mRNA-S+N induced a more robust effect, leading to complete viral control with no detection of the viral RNAs on 4 DPI (mRNA-S vs. mRNA-S+N: *p* < 0.0001 on 4 DPI) (Fig. 3C). We previously showed that the stronger protection against an Omicron variant (BA.1) by mRNA-S+N relative to mRNA-S alone was not due to the difference in total mRNA or LNP doses^11^. Lastly, vaccination with mRNA-S alone and mRNA-S+N both protected the hamsters from weight loss, with no significant difference detected between the two vaccine groups (Fig. 3D).

### mRNA-S+N vaccination induces strong protection against SARS-CoV-2 Omicron BQ.1 in hamsters

**Fig. 4:**
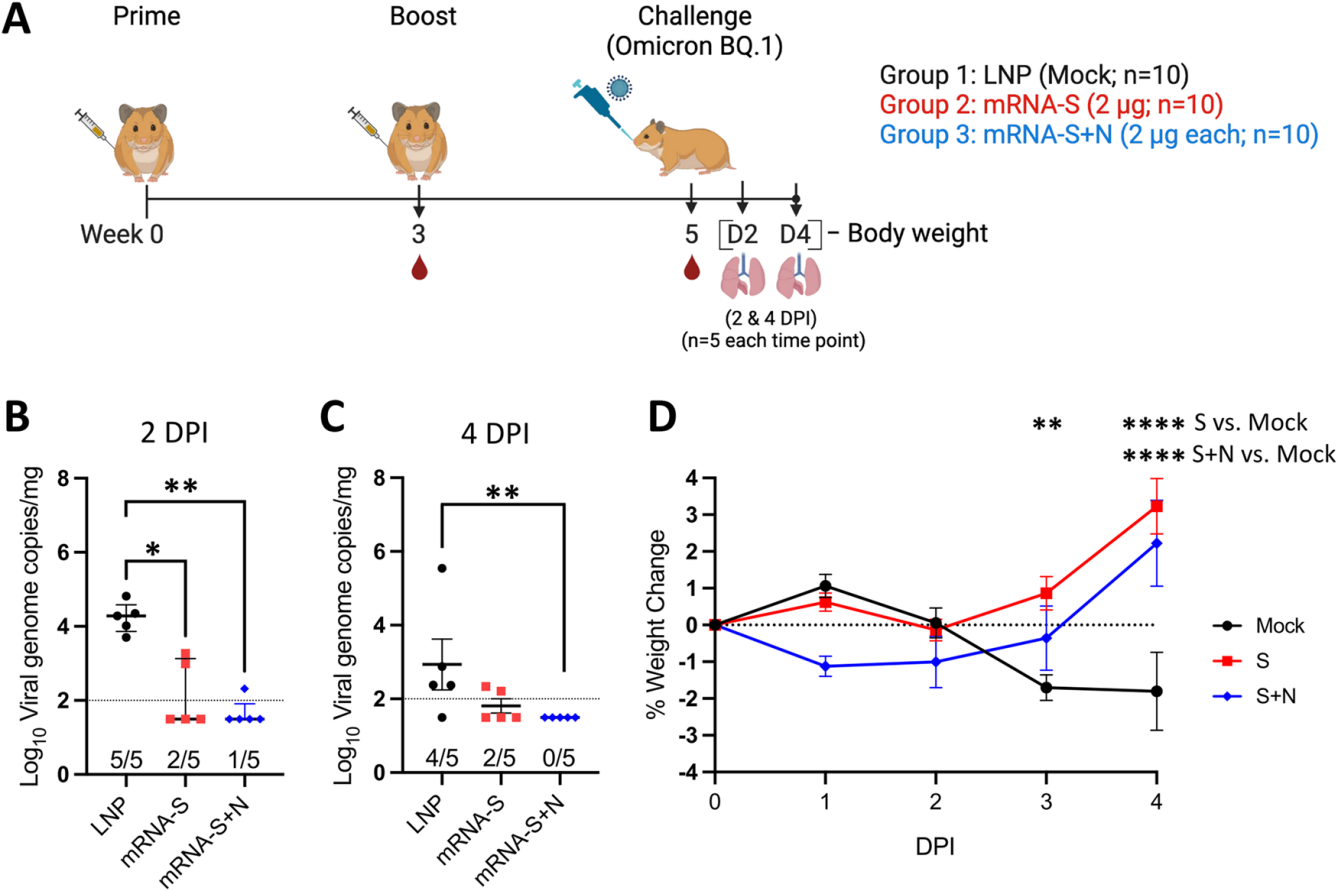
Dual mRNA-S+N vaccination protects hamsters from Omicron BQ.1. **A** Experimental design and timeline (Created with BioRender.com). Three groups of hamsters (*n* = 10/group) were vaccinated intramuscularly with empty LNP (mock), mRNA-S (2 μg), or mRNA-S+N (2 μg for each mRNA) at weeks 0 and 3, followed by intranasal challenge with SARS-CoV-2 Omicron BQ.1 (2 × 10^4^ pfu) at week 5. Lungs were harvested at 2 (**B**) and 4 (**C**) DPI (*n* = 5 at each time point) for quantification of viral RNA copies by RT-qPCR. **D** Hamster body weights were monitored from 0 to 4 DPI. In (**B**, **C**), symbols represent individual animals, midlines represent the median, error bars represent the interquartile range, and the dashed line represents the lower limit of detection (LOD). The number of animals with viral loads above the LOD is noted. Log_10_ normalized data was compared among the three groups by Kruskal-Wallis test. In (**D**), symbols represent the mean, error bars represent the standard deviation, and the dashed line highlights 0% body weight change. Weight change was compared by 2-way ANOVA followed by Tukey’s multiple comparisons test (two factors; hamsters’ weight and time). *p < 0.01, **p < 0.01, ****p < 0.0001.

To further determine the breadth of protection by mRNA-S+N, we next examined efficacy of mRNA-S and mRNA-S+N against BQ.1 in the hamster model. BQ.1, derived from BA.5, emerged with additional spike mutations that contributed to its strong immune evasion and efficient transmission^13,28^. Hamsters were vaccinated at weeks 0 and 3 before intranasal challenge with BQ.1 (2 × 10^4^ pfu) at week 5 (Fig. 4A). Lungs were harvested at 2 (*n* = 5) and 4 (*n* = 5) DPI with hamster body weights monitored throughout the course of the experiment (Fig. 4A). Compared to BA.5 (Fig. 3) and earlier variants (BA.1 and Delta)^11^, BQ.1 appears to replicate to lower levels in hamsters and with faster clearance by 4 DPI (Fig. 4B, C). Similarly, body weight data indicated that nonvaccinated (mock) hamsters showed marginal weight loss through 4 DPI (Fig. 4D). This is consistent with the observations that BQ.1 is moderately pathogenic compared to BA.5^13,29^. Vaccination with mRNA-S+N led to complete viral control with no detection of viral RNA in four out of five hamsters at 2 DPI (Fig. 4B) and five out of five hamsters at 4 DPI (Fig. 4C). Both mRNA-S and mRNA-S+N protected hamsters from the mild weight loss resulting from BQ.1 infection with no significant difference detected between the two vaccine groups (Fig. 4D).

### mRNA-S+N induces binding antibodies that manifest no detectable neutralizing activities against BA.5 and BQ.1 variants

**Fig. 5:**
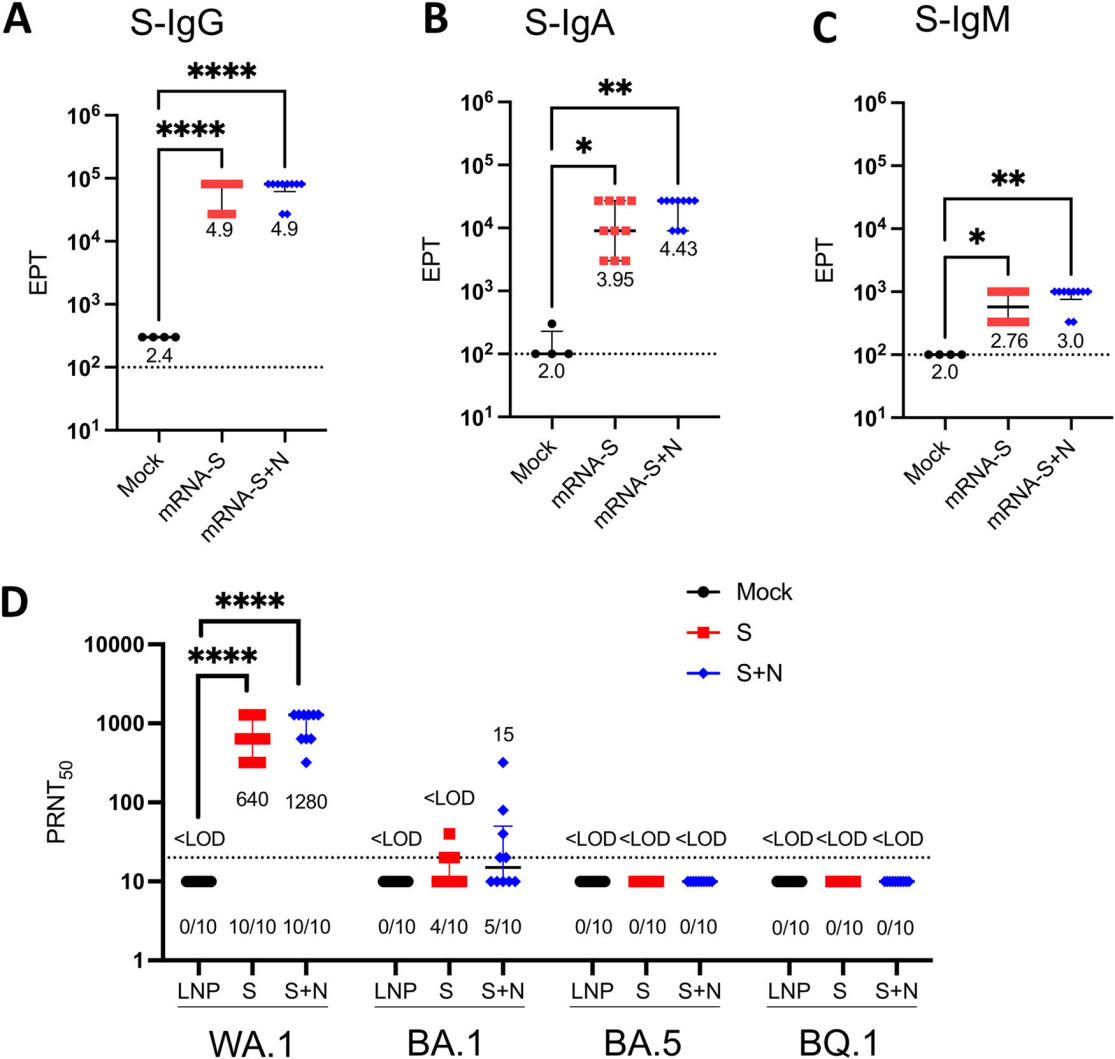
Serum antibody response in hamsters following mRNA vaccination. S-specific binding IgG (**A**), IgA (**B**), and IgM (**C**) endpoint titers (EPTs) in the hamster sera at week 5 after immunization (*n* = 4 for LNP; *n* = 10 for mRNA-S or mRNA-S+N). Log_10_ normalized EPTs for each group is indicated. **D** Neutralization of the hamster sera (*n* = 10 for all three groups) against SARS-CoV-2 WA.1, BA.1, BA.5, and BQ.1 as measured by PRNT_50_. Data are presented as median with interquartile range. Dotted lines in each plot indicates LOD for each assay. Number of animals in each group with neutralizing titers above the LOD is noted in (**D**). Data were compared among the three groups by Kruskal-Wallis test. *p < 0.05, **p < 0.01, ****p < 0.0001.

In the above hamster experiments (Fig. 3, 4), serum samples were collected prior to viral challenge (two weeks after booster) for analysis of vaccine-induced antibody responses (Fig. 5). Binding antibody endpoint titers (EPTs) were determined by ELISA. Compared to the mock control, both mRNA-S alone and mRNA-S+N induced significant levels of S-specific binding antibodies in sera (Fig. 5A–C). Median IgG EPTs for both mRNA-S and mRNA-S+N groups were 10^4.9^ (Fig. 5A). Other than IgG, serum IgA and IgM were also detectable. Median IgA EPTs for mRNA-S and mRNA-S + N were 10^3.95^ and 10^4.43^, respectively (Fig. 5B), and median IgM EPTs for mRNA-S and mRNA-S+N were 10^2.76^ and 10^3^, respectively (Fig. 5C). These serum samples were also examined for neutralization against SARS-CoV-2 WA.1, BA.1, BA.5, and BQ.1 using the plaque-reduction neutralization test (PRNT) with corresponding live virus. While the sera of hamsters immunized with mRNA-S and mRNA-S+N showed strong neutralizing activity against the ancestral WA.1 strain (Fig. 5D), their neutralizing activities against BA.1 were substantially reduced: 4/10 samples had weakly detectable neutralization in the mRNA-S alone group and 5/10 weakly detectable neutralization in the mRNA-S+N group. Notably, neutralizing activities of these sera against BA.5 and BQ.1 variants were completely lost, with all samples showing undetectable neutralization activity for these variants (Fig. 5D). These data are consistent with the observations that BA.5 and BQ.1 manifest strong immune escape from the ancestral S-based vaccine induced neutralization. The data also show that the strong viral control by mRNA-S+N in the hamster lungs is occurring even in the absence of detectable neutralizing antibodies, indicating a role of cellular immunity in mRNA-S+N induced protection against variants as described previously^11^.

### mRNA-S+N vaccination induces strong respiratory T cell responses in the lungs and bronchoalveolar lavage

After showing that mRNA-S+N vaccine induced complete viral control in the hamster lungs in the absence of detectable neutralizing antibodies, we next examined respiratory immune responses induced by mRNA-S+N as compared to mRNA-S alone. For this, we utilized the C57BL/6 mouse model and the available S- (S_539–546_: VNFNFNGL) and N- (N_219–227:_ LLLDRLNQL) specific MHC-I tetramers. Similar to N_219–227_ (Fig. 2), S_539-546_ (VNFNFNGL) is a highly dominant CD8 T cell epitope within the S protein that was predicted based on bioinformatic analysis and later validated in the context of both infection and vaccination^30–32^. Multiple vaccine studies have confirmed a protective role of the CD8 T cell response induced against this particular S epitope^33,34^.

**Fig. 6:**
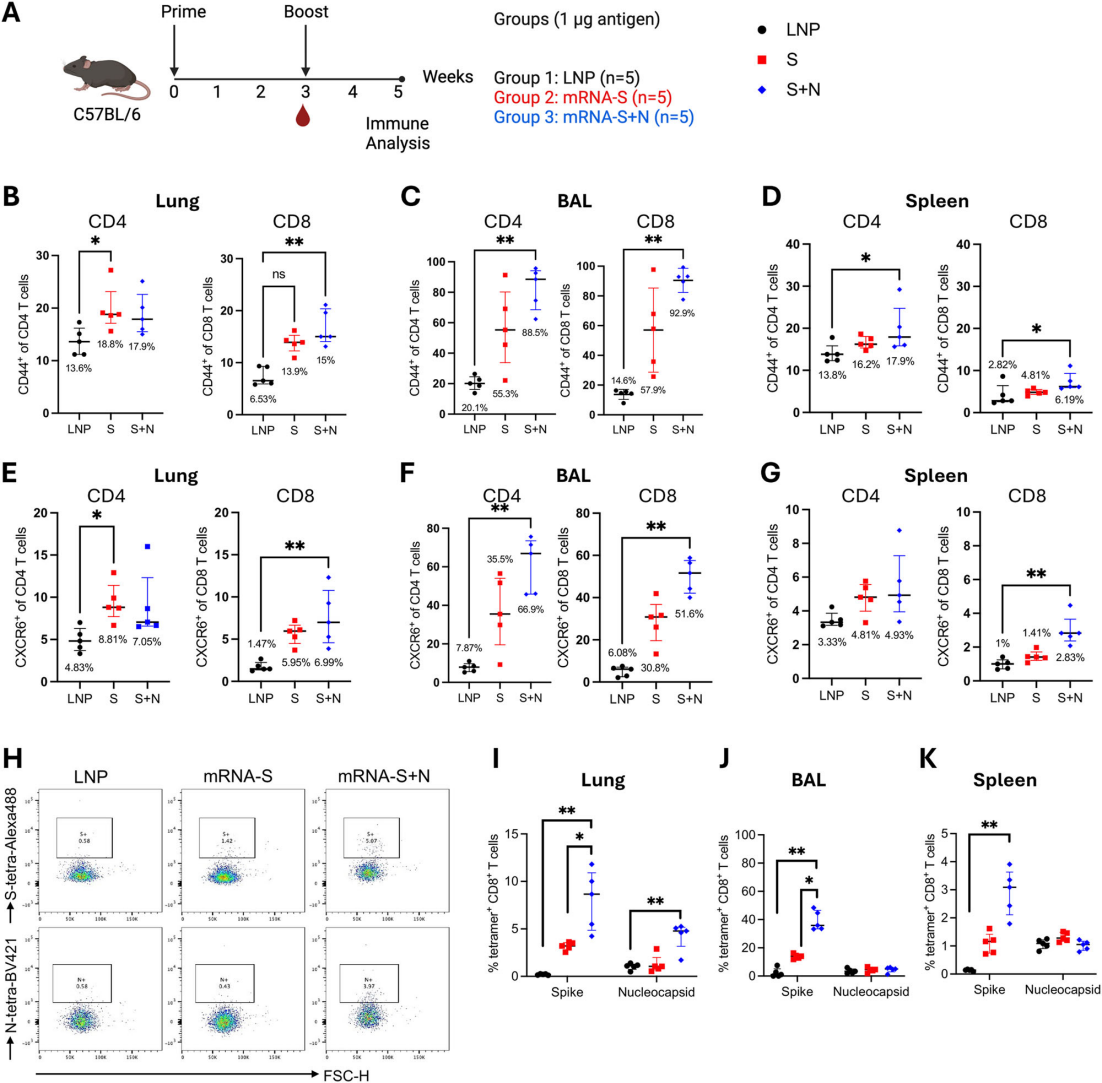
Respiratory T cell response following different mRNA vaccination. **A** Mouse experimental design and timeline (Created with BioRender.com). Three groups of C57BL/6 mice (*n* = 5/group) were intramuscularly vaccinated with either empty LNP (mock), mRNA-S vaccine (1 μg), or mRNA-S+N vaccine (1 μg for each mRNA) at week 0 and 3. Two weeks after final dose (week 5), mice were euthanized and immune analyses were performed. Analysis of activated T cells in the lungs (**B**), BAL (**C**), and spleens (**D**) of mice. Expression of CD44 on CD4 and CD8 T cells was examined by flow cytometry and shown as percent CD44^+^ of the parental population. Expression of CXCR6 on CD4 and CD8 T cells in the lungs (**E**), BAL (**F**), and spleens (**G**) was examined by flow cytometry and shown as percent CXCR6^+^ of the parental population. **H** Representative flow cytometry plots for S- and N-tetramer staining of CD8 T cells from LNP, mRNA-S, and mRNA-S+N vaccinated mice. Tetramer^+^ (S- and N-epitope specific CD8 T cells) in the lungs (**I**), BAL (**J**), and spleens (**K**) of mice. Data are presented as median and interquartile range. Kruskal-Wallis test was used for statistical comparison among the three groups. *p < 0.05, **p < 0.01.

Three groups of C57BL/6 mice (*n* = 5 per group) were vaccinated (IM) with empty LNP (mock), mRNA-S alone (1 μg), or mRNA-S+N (1 μg per mRNA) at week 0 and 3, followed by analysis of vaccine-induced immune responses in the lungs and BAL (Fig. 6A). T cell responses in the spleen were also examined for comparison. The gating strategies to identify T cell populations and tetramer^+^ CD8 T cells are shown in Supplementary Fig. 1, Supplementary Fig. 4, and the main figures.

In the lungs, compared to mock control, both mRNA-S and mRNA-S+N elicited slightly higher levels of total activated (CD44^+^) CD4 T cells, although only the mRNA-S group reached statistical significance (*p* < 0.05 for mock versus mRNA-S) (Fig. 6B, left). A significant increase in the frequency of total activated CD8 T cells in the lungs of mRNA-S+N-vaccinated mice (median: 15%) was observed when compared to the mock group (median: 6.53%) (*p* < 0.01) (Fig. 6B, right). In the BAL, a stronger effect was observed. Compared to mock control, mRNA-S+N vaccine elicited substantially higher levels of activated CD4 (Mock versus mRNA-S+N: 20.1%, 88.5%; *p* < 0.01) and CD8 (Mock versus mRNA-S+N: 14.6%, 92.9%; *p* < 0.01) T cells (Fig. 6C). Compared to mRNA-S+N, mRNA-S alone induced weaker CD4 and CD8 T cell activation in the BAL and no statistical significance was detected between the two vaccine groups (Fig. 6C). In contrast, in the spleen, we only noted a modest increase in CD4 and CD8 T cell activation in the mRNA-S+N group compared to mock control (Fig. 6D). In all these compartments, no significant difference in the frequency of total CD4 and CD8 T cells was observed among the groups (Fig. S6).

We next examined frequencies of T cells positive for CXCR6 in the lungs, BAL, and spleen (Fig. 6E–G). A pattern similar to the activated T cells was revealed. Compared to the mock control, mRNA-S+N elicited significantly higher levels of CXCR6^+^ CD8 T cells in the lungs (1.47% versus 6.99%; *p* < 0.01) (Fig. 6E) as well as in the BAL (6.08% versus 51.6%, *p* < 0.01) (Fig. 6F). In the BAL, mRNA-S+N also induced a markedly higher level of CXCR6^+^ CD4 T cells when compared to the mock control (7.87% versus 66.9%, *p* < 0.01) (Fig. 6F). These data indicate lung-homing potential of the induced T cells by mRNA-S+N. In contrast, mRNA-S alone only elicited a trend towards increase in CXCR6^+^ CD4 and CXCR6^+^ CD8 T cells in the BAL when compared to the mock control (Fig. 6F). As anticipated, lower levels of CXCR6^+^ T cells were observed in the spleens when compared to those in the BAL and lung following mRNA-S or mRNA-S+N vaccination (Fig. 6G).

Antigen-specific CD8 T cells in these compartments were measured (Fig. 6H–K). Representative flow cytometry plots for S- and N-tetramer staining showed that mRNA-S alone elicited S-specific, but not N-specific, CD8 T cells, while mRNA-S+N vaccine elicited both S- and N-specific CD8 T cells, supporting the specificity of the tetramer staining (Fig. 6H). We observed that, compared to mRNA-S alone, mRNA-S+N vaccine elicited higher levels of S-specific CD8 T cells in the lungs (*p* < 0.05) (Fig. 6I). A more profound effect was observed in the BAL, where mRNA-S+N induced much higher levels of S-specific CD8 T cells (~36%) than mRNA-S (~14%) (Fig. 6J). Of note, while mRNA-S+N also elicited detectable N-specific CD8 T cells in the lungs (Fig. 6I), their levels in the BAL were low (Fig. 6J). Lastly, only low levels of S-epitope specific CD8 T cells were detected in the spleen after mRNA-S+N vaccination (Fig. 6K). Together, these data indicate that mRNA-S+N elicits strong respiratory T cell responses, especially S-specific CD8 T cells, after IM mRNA immunization. The data also reveal that the presence of N for co-immunization has some synergistic effect and augments S-specific T cell response in the respiratory tract, a finding also observed for systemic immunity in our previous study^11^.

Vaccine-induced binding antibodies in the BAL were also examined. Compared to mock control, both vaccines induced significant levels of S-specific binding IgG (Fig. S7). As expected, only mRNA-S+N vaccination resulted in the production of both S- and N-specific binding IgG in the BAL (Fig. S7), whereas mRNA-S alone only elicited S-specific binding IgG. Unlike IgG, there was a lack of detectable antigen-specific IgA in the BAL, consistent with the result of mRNA-N vaccination (Supplementary Fig. 5) as well as with a previous study reporting limited mucosal IgA production following IM mRNA immunization^15^.

## Discussion

The emergence of novel SARS-CoV-2 variants has greatly reduced effectiveness of the first-generation COVID-19 vaccines^35^. Our previous study reported the efficacy of the dual mRNA-S+N vaccine against Delta and Omicron BA.1 strain in animal models^11^. The present study demonstrates that mRNA-S+N, based on the ancestral viral sequence, induces robust protection against the more immune evasive Omicron subvariants, BA.5 and BQ.1, in the absence of detectable neutralizing antibodies. Additionally, in contrast to our previous study which examined vaccine-induced systemic immunity^11^, this study investigates mRNA vaccine-induced respiratory immunity and shows that peripheral immunization with mRNA-S+N effectively elicits respiratory S- and N-specific CD8 T cell responses in the animal lungs and BAL at the time examination (2 weeks after booster). The data together support mRNA-S+N as a promising pan-SARS-CoV-2 vaccine approach and provide additional immunological insights in mechanisms of protection conferred by mRNA-S+N against immune-escaping variants.

There are ongoing efforts to develop a next-generation, broadly protective SARS-CoV-2 vaccine against current and emerging variants. Targeting conserved regions of the virus, e.g., N protein, in addition to the S protein, is considered an attractive strategy for pan-COVID-19 vaccine development^36,37^. In support, our analysis reveals that N protein has higher sequence conservation among the different SARS-CoV-2 variants (BA.1, BA.5, BQ.1., and Wuhan-Hu-1) as compared to the S protein (Table 1). N is a structural protein of SARS-CoV-2 with abundant expression in infected cells and plays a role in the coronavirus life cycle, including viral genome packaging and immune regulation^38^. Notably, the N-induced memory T cell response is cross-reactive^39^. In this study, we further demonstrate that mRNA-N alone induces incomplete but significant control of Omicron BA.5 infection in hamster lungs. The data are consistent with our previous report showing a modest protective effect of this vaccine alone against mouse-adapted SARS-CoV-2 and Delta variant^11^.

Our study also determines the efficacy of dual mRNA-S+N vaccine against newly emerged immune-escaping Omicron subvariants BA.5 and BQ.1. Compared to the early variants, BA.5 possesses additional changes (69-70del, L452R, and F486V) and a reversion (R493Q) in the S protein (Table 1), which contribute to its enhanced fusogenicity and infectivity^22^. Studies in rodents showed that BA.5 has superior viral fitness to that BA.2^40^. In addition, when the present study was conducted, the BQ.1 variant which bears additional mutations in its S protein (i.e., N460K, R346T, and K444T) (Table 1) had emerged as a dominant circulating strain with enhanced transmissibility and immune evasion^35,41,42^. In line with these observations, our data showed that while the sera of mRNA-S or mRNA-S+N-immunized hamsters manifested strong neutralization of the prototypical WA.1 strain, and even the Delta strain^11^, their neutralizing activities were greatly reduced against Omicron BA.1 (only a few animals in each vaccine group showed weak neutralization) and were completely lost against BA.5 and BQ.1 (Fig. 5D)^43,44^. However, even in the absence of detectable neutralizing antibodies, we still observed complete viral control in hamster lungs by mRNA-S+N against both BA.5 and BQ.1. Indeed, our previous study using in vivo CD8 cell depletion did support a role of T cell immunity in protection^11^. Despite these data, immune mechanisms other than T cell immunity, such as other effector functions of antibodies induced by S and N^23,45,46^, should not be excluded and warrant further investigation. Nevertheless, our data confirmed the breadth of protection conferred by mRNA-S+N, irrespective of S-based mutations, and support the idea that vaccine approaches inducing robust, cross-protective cellular immunity should be pursued^47,48^.

Respiratory immunity is important for protection against SARS-CoV-2 infection. Studies on induction of respiratory immunity by peripheral mRNA vaccination have reported inconsistent results. Some studies reported minimal T cell and antibody responses in the airway mucosa after peripheral vaccination^15,16^, while others showed detectable mucosal IgA along with CD4 and CD8 T cells in vaccinated individuals^17,18^. In our study, we analyzed total and antigen-specific T cells in the lungs and BAL of the immunized mice. We demonstrated that both S and N epitope-specific CD8 T cells were detectable following IM mRNA-S+N vaccination in the lungs and/or BAL. This induction of a robust respiratory T cell response correlates with the strong control of BA.5 and BQ.1 by mRNA-S+N in the absence of neutralizing antibodies. Thus, our data favor that peripheral mRNA vaccination could induce T cell responses in the airway, although it remains unclear if these T cell responses are durable, since we only examined the response at the peak immunogenicity following vaccination (2 weeks after booster). Our analysis of antibodies showed that only binding IgG was detectable in the BAL. Lack of detection of mucosal IgA following IM mRNA vaccination in our study is consistent with previous reports^15,16^ and supports the need for a mucosal booster to further improve respiratory immunity after peripheral mRNA vaccination.

The present study utilized MHC-I tetramer staining to identify antigen-specific CD8 T cells in the mouse lungs and BAL. Compared to intracellular cytokine staining (ICS), tetramer staining requires fewer cells and no peptide restimulation, thus making it less challenging to measure antigen-specific T cells in samples of low cell numbers (e.g., BAL), although tetramer staining only identifies T cells specific to single epitope. In our study, two MHC-I epitopes (N_219-227_: LLLDRLNQL; S_539-546_: VNFNFNGL) were used to detect N- and S-specific CD8 T cells in mouse BAL/lungs. Both are dominant CD8 T cell epitopes within the SARS-CoV-2 N and S protein, respectively. The two epitopes were initially predicted by bioinformatical analysis^8^ and subsequently validated in humans and mice^24,25,30–32^. In particular, the protective role of CD8 T cell responses against the single S_539-546_ epitope was confirmed in multiple SARS-CoV-2 vaccine studies^33,34^. Consistent with these reports, our study shows that IM mRNA-S+N vaccination elicits both S_539-546_ and N_219-227_ epitope-specific CD8 T cell populations in the mouse airway (Fig. 6). It is interesting to note that the magnitude of S_539-546_-specific CD8 T cell response is markedly higher than N_219-227_-specific CD8 T cell response in the mouse BAL/lungs, although the dose of mRNA-S and mRNA-N used in the dual immunization regimen (mRNA-S+N) is identical. This is likely due to multiple reasons. First, the co-immunization of N augments S-specific immunity, but not vice versa. This pattern is particularly evident in the CD8 T cell responses, as seen here and in our previous study^11^. The underlying mechanisms are not yet clear. Second, the S_539-546_ epitope may be more dominant within the S protein than the N_219-227_ epitope within the N protein following mRNA vaccination. Also, it is possible that CD8 T cells specific to other N epitopes are also induced by mRNA vaccines but not detectable using the N_219-227_ tetramer staining.

Other than CD8 epitopes, a number of CD4 T cell epitopes within the SARS-CoV-2 S and N proteins were also identified^8,49^, despite their protective roles in vaccination are less clear. Our study examined respiratory CD4 T cells with activation (CD44) or lung-tissue residency (CXCR6) phenotype and showed that IM mRNA-S+N vaccination elicits high levels of activated and CXCR6^+^ CD4 T cells, especially in the BAL (Fig. 6C, F). The present study did not measure respiratory antigen-specific CD4 T cells, largely due to the current lack of tetramers for these CD4 epitopes. However, our previous study used ICS assay and showed the induction of robust systemic antigen-specific CD4 T cells by mRNA-S+N vaccine in BALB/c mice^11^. We thus speculate that peripheral mRNA vaccination should also elicit antigen-specific CD4 T cells in BAL/lungs. Nevertheless, a more comprehensive characterization of the respiratory CD4 and CD8 T cell responses specific to the full S and N protein in the BAL/lungs following mRNA vaccination is needed in future studies.

The present study has several limitations. First, in the respiratory immune analysis, single cell suspensions were prepared from the whole lung tissue without discriminating immune cells from circulating blood, raising the concern that the total and antigen-specific T cells identified in the lungs could also contain contaminated cells from the blood. However, our analysis of T cells in the BAL supports that mRNA vaccination induces respiratory T cell responses in the airway. Second, while we show the induction of antigen-specific T cells in the lung and BAL at peak immunogenicity, whether they are long-lasting, tissue-resident T cells and can confer durable protection remain unclear. Lastly, with the vast majority of the human population having received the S-based vaccines or being naturally infected with SARS-CoV-2, mRNA-N as a booster component for inducing a broad protection in the context of pre-existing immunity should be investigated, which could expand the potential utility of this vaccine candidate.

## Materials and methods

### Study design

The aim of the present study was to investigate efficacy of mRNA-N and mRNA-S+N against newer SARS-CoV-2 Omicron variants and the respiratory immunity induced by these mRNA vaccines in animal models. Evaluation of vaccine efficacy against Omicron BA.5 and BQ.1 challenges were conducted in Syrian hamsters, and analysis of vaccine-induced respiratory immunity was conducted in C57BL/6 mice. The animal study protocols were approved by the Institutional Animal Care and Use Committee at the University of Texas Medical Branch (Protocol numbers: 1703020, 2009087). All animal experiments were carried out following the recommendations in the Guide for the Care and Use of Laboratory Animals of the National Institutes of Health. During the studies, all animals were monitored by animal resources center or laboratory staff daily. The number of animals utilized per group was determined by a power analysis for a medium effect size prior to the onset of the study and all data points were utilized for analysis. All animals were allowed a minimum of 3 days to acclimate to their environment before study onset and anesthetized with 1–5% isoflurane via vaporizer prior to each procedure apart from weights. Mice were humanely euthanized with CO_2_ asphyxiation followed by cervical dislocation. Hamsters were humanely euthanized with CO_2_ asphyxiation followed by bilateral thoracotomy. The study design was not blinded to researchers or staff at the animal facility, and animals were randomly assigned to each group.

### mRNA synthesis and LNP formulation

mRNA synthesis and LNP formulations were prepared as published previously^11^. Briefly, mRNA-N (full-length) and mRNA-S (prefusion-stabilized S protein with two proline mutations; mRNA-S-2P) were synthesized by using the T7 RNA polymerase (MegaScript, Thermo Fisher Scientific) in vitro transcription kit. The sequences are based on the ancestral SARS-CoV-2 Wuhan-Hu-1 strain (GenBank MN908947.3). Uridine triphosphate was replaced with one-methylpseudouridine (m1)-5′-triphosphate. For improved protein expression, polyadenylated tails were added to the ends of modified mRNAs. ScriptCap m7G capping system and ScriptCap 2′-O-methyltransferase kit were used to cap in vitro transcribed mRNAs, which were then underwent cellulose-based purification. As previously reported, the mRNAs were formulated into LNPs using an ethanolic lipid mixture of ionizable cationic lipid and an aqueous buffer system. mRNA-LNPs were prepared in accordance with RNA concentrations (1 µg/µl) and stored at −80 °C prior to animal immunizations. Protein expression in cells after mRNA-N and mRNA-S LNP treatment was confirmed in our previous publication^11^.

### Animal immunizations and SARS-CoV-2 challenge

mRNA vaccine-induced immune response was evaluated in mice. 6-week-old female C57BL/6 mice purchased from the Jackson Laboratory (strain no. 000664) were randomly assigned into four groups (*n* = 5 per group) that were respectively immunized IM (thigh muscles of the hind limb) with 50 µl of either empty LNP (mock), mRNA-S (1 μg), mRNA-N (1 μg), or mRNA-S+N (1 μg per mRNA) at week 0 (prime) and week 3 (boost). 2 weeks after booster vaccination (week 5), all mice were euthanized (CO_2_ and physical dislocation) for terminal blood collection and tissue harvests. Lung and BAL samples were collected for analysis of vaccine-induced respiratory immune responses. In some experiments, sera and spleen samples were also collected to analyze vaccine-induced systemic immune response for comparison.

mRNA vaccine efficacy was evaluated in hamsters. 3-week-old male golden Syrian hamsters purchased from Envigo (strain HsdHan: AURA, catalog no. 8901M) were randomly assigned into four groups (*n* = 10 per group). They were respectively vaccinated IM (thigh muscles of the hind limb) with 100 µL of either LNP (mock), mRNA-S (2 µg), mRNA-N (2 µg), or combined mRNA-S+N (2 µg per mRNA) at weeks 0 and 3. Two weeks after booster (week 5) and prior to viral challenge, all hamsters were subject to serum collection under anesthesia. The sera were used to measure vaccine-induced antibodies. Hamsters were then transferred to ABSL-3 facility and intranasally challenged with either SARS-CoV-2 Omicron BA.5 (2 × 10^4^ pfu in 100 µl/hamster) or BQ.1 (2 × 10^4^ pfu in 100 µl/hamster) under anesthesia. At 2- and 4 DPI, five hamsters of each group (for each time point) were euthanized and the lung tissues were collected for evaluating vaccine-induced protection. Hamster body weights were also monitored daily from the day of viral challenge to 4 DPI.

### Spike-specific binding antibodies in hamster sera

Vaccine-induced S-specific binding antibody titers (IgG, IgA, and IgM) in the hamster sera were determined by ELISA. Plates (Greiner Bio-One) were coated with recombinant S protein (1 µg/ml; 40589-V08B1, Sino Biological) overnight at 4°C. Plates were washed three times and then blocked with blocking buffer [8% fetal bovine serum (FBS) in Dulbecco’s PBS (DPBS)] for 1 h at 37 °C, followed by washing and incubation at 37 °C for 2 h with serially diluted serum samples (initial dilution 1:100, followed by 3-fold serial dilution) in blocking buffer at 50 µl per well. Plates were washed again and incubated with HRP-conjugated anti-hamster IgG (1:1000; Southern Biotech; 6060-05), anti-hamster IgA (1:1000; Brookwood Biomedical; sab3003a), or anti-hamster IgM (1:500; Brookwood Biomedical; sab3003m) secondary antibodies for 1 h (IgG) or overnight (IgA and IgM) at 37 °C. After incubation, plates were washed and developed using TMB 1-Component Peroxidase Substrate (Thermo Fisher Scientific), followed by termination of reaction using the 2 N HCl solution. Plates were read at 450 nm wavelength within 15 min by using a Microplate Reader (BioTek). Binding antibody EPTs were calculated.

### Spike- and nucleocapsid-specific binding antibodies in mouse bronchoalveolar lavage

S- and N-specific binding IgG and IgA in mouse BAL were measured by ELISA as described above except the BAL samples underwent 1:1 dilution.

### Flow cytometry analysis

Single cell suspensions prepared from lung, BAL, and spleen were directly stained for viability (65-0866-14, Invitrogen; Fixable Viability Dye eFluor 506) and surface markers including CD3- BUV805 (741982, BD Biosciences; clone 17A2), CD4-APC/Fire 570 (100460, BioLegend; clone GK1.5), CD8-BV570 (100740, Biolegend; clone 53-6.7), CD44-PerCP (103036, Biolegend; clone IM7), CXCR6-BV711 (151111, Biolegend; clone SA051D1). To detect S- and N-specific CD8 T cells, samples were incubated with S_539–546_ MHC-I tetramer-Alexa488 (H-2Kb, 1:500 dilution) and N_219–227_ MHC-I tetramer-BV421 (H2-Db, 1:500 dilution) (NIH Tetramer Core) for 30 min at room temperature. Cells were washed and then acquired on a flow cytometer FACSymphony A5 (BD Biosciences). Data were analyzed using FlowJo (Flowjo LLC).

### Quantification of viral RNA by RT-qPCR

Hamster lungs were collected in maintenance medium (2% FBS in DMEM with 1% pen-strep) and subjected to homogenization for 1 min. Debris was pelleted by centrifugation for 5 min at 16,000 *g*. Following homogenization, the supernatants of tissue homogenates were combined with a fivefold volume of TRIzol LS Reagent (Thermo Fisher Scientific). Extraction of viral RNAs was performed in accordance with the instructions provided by the manufacturer. The final RNA solutions were stored at −80 °C until use for RT-qPCR. Viral RNA copies were determined in the lungs by using the one-step RT-qPCR kit (Bio-Rad, 1725151) on CFX Connect Real-Time PCR Detection System (Bio-Rad). SARS-CoV-2 E gene primers (forward, 5′-GGAAGAGACAGGTACGTTAATA-3′; reverse, 5′-AGCAGTACGCACACAATCGAA-3′) were used. The PCR reaction was composed of primers (10 μM), RNA sample (2 μl), iTaq universal SYBR Green 1-step reaction mix (2×; 10 μl), iScript reverse transcriptase (0.25 μl), and molecular grade water, for a total volume of 20 μl. The PCR cycling conditions were as follows: 95 °C for a duration of 3 min, followed by 45 cycles of 95 °C for 5 s and 60 °C for 30 s. A standard curve was included in each RT-qPCR analysis, utilizing an RNA standard that was synthesized in vitro. This RNA standard consisted of 3839 base pairs and encompassed genomic nucleotide locations 26,044 to 29,883 of the SARS-CoV-2 genome. By using the standard curve we determined the absolute number of viral RNA copies present in the lung tissue^11^.

### Virus neutralization analysis

Serum neutralizing activity was examined using PRNT assay^11^. The experiments were carried out on Vero E6 cells (ATCC, CRL-1586) with the SARS-CoV-2 wild-type or Omicron variants. Briefly, serum samples were heat-inactivated (at 56 °C for 30 min) and serially diluted twice (1:10 initial dilution and then twofold serial dilutions), followed by incubation for 1 h at 37 °C with wild-type SARS-CoV-2 (USA-WA1/2020), BA.5, or BQ.1, respectively. The above mixtures were added to monolayers of Vero E6 cells in 6-well plates for incubation at 37 °C for 1 h. After incubation, a 2 ml of semisolid overlay medium (minimum essential medium containing 1.6% agarose, 2% FBS, and 1% penicillin-streptomycin) was added to the cells, and then incubated for 48 h at 37 °C. After incubation, monolayer cells were stained with 0.03% liquid neutral red for 3–6 h. A manual counting method was used to count the number of plaques and PRTN50 was calculated.

### Statistical analysis

Statistical analysis was performed using GraphPad Prism 10.1.1 software. Data were presented as median ± IQR or mean ± standard deviation, as denoted in the figure legends. Statistical comparison among groups was performed using Mann-Whitney test, Kruskal-Wallis test, or two-way ANOVA with Tukey’s multiple comparison test where appropriate as denoted in the figure legends. Two-tailed *p* values were denoted, and *p* < 0.05 were considered significant. All analyses were conducted assuming a 95% confidence interval.

## Supplementary information

Supplementary Information

## Data Availability

The datasets generated and analyzed in this study are presented in the manuscript and the supplementary files. Original raw data are also available from the corresponding authors upon request.
