# Supplementary material for: Broad protection and respiratory immunity of dual mRNA vaccination against SARS-CoV-2 variants
Source: NPJ Vaccines. 2024 Sep 4;9:160. doi: 10.1038/s41541-024-00957-2 (PMC11374988; doi:10.1038/s41541-024-00957-2)
Supplement: Supplementary file 1 — Supplementary Information [file 41541_2024_957_MOESM1_ESM.pdf]

Supplemental Figures

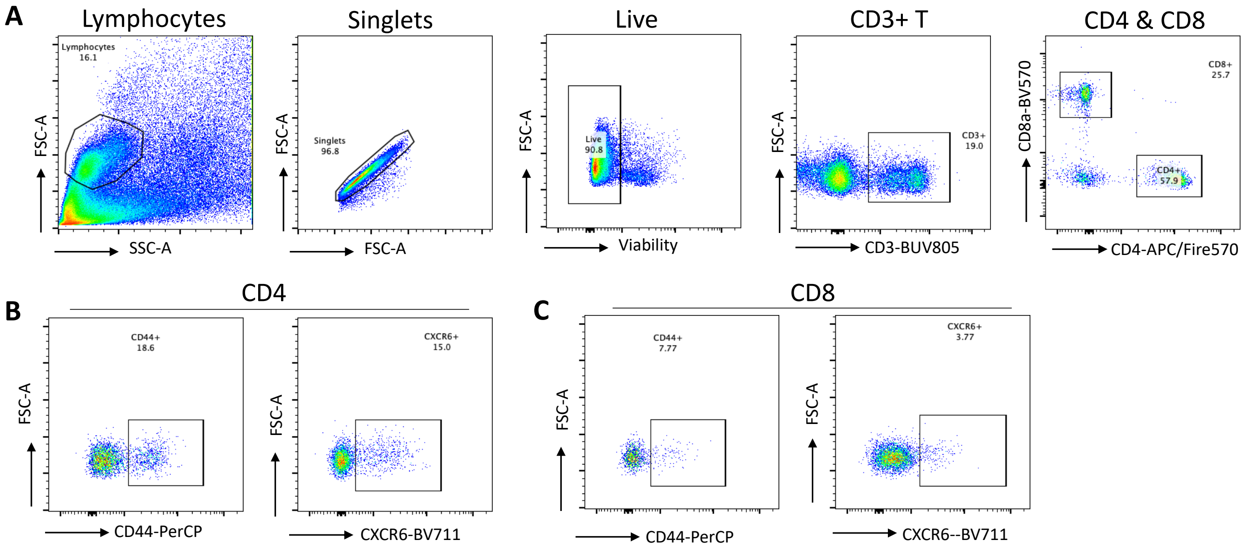

Supplementary Figure 1. Gating strategy for T cell analysis in the lungs. Mice were vaccinated intramuscularly with mRNA vaccine on a prime-boost schedule. Two weeks after the booster dose (week 5), mouse lungs were harvested for preparation of single cell suspensions. Cells were stained and subjected to flow cytometric analysis. **(A)** Lymphocytes were gated based on FSC-A and SSC-A, followed by gating on singlets. Fixable viability dye was used to differentiate live and dead cells, followed by gating on CD3 T cells. CD3 T cells were further gated as CD4 and CD8 T cell subsets. Next, CD44 as a marker of T cell activation, and CXCR6 as a marker of cells with tissue residency potential, in CD4 **(B)** and CD8 **(C)** T cells were gated.

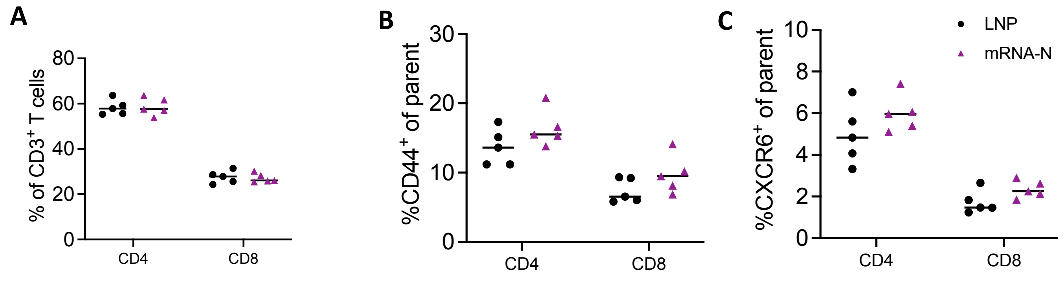

Supplementary Figure 2. T cell analysis in the lungs following empty LNP or mRNA-N vaccination. Mice (n=5 per group) were vaccinated intramuscularly with mRNA on a prime-boost schedule at week 0 and 3. Two weeks after booster dose (week 5), mouse lungs were harvested for preparation of single cell suspensions. Cells were stained and subjected to flow cytometric analysis. **(A)** Percentages of CD4+ and CD8+ in CD3+ T cell population. **(B)** Percentages of activated CD4 or CD8 T cells based on CD44 expression. **(C)** Percentages of CXCR6+ CD4 and CXCR6+ CD8 T cells. Horizontal line in each plot indicates median.

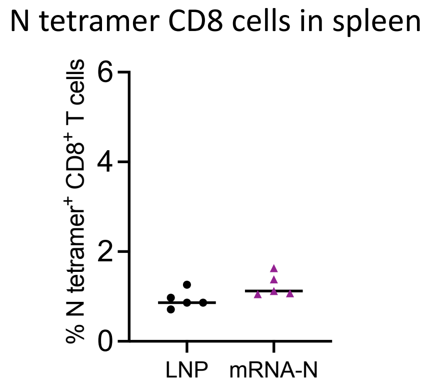

Supplementary Figure 3. N-tetramer+ CD8 T cells in the spleen. Analysis of tetramer positive, N-specific T cells in the spleen by flow cytometry. Frequency of N-specific $CD8^+$ T cells in the spleens was compared between mock and vaccine group. Horizontal lines indicate median.

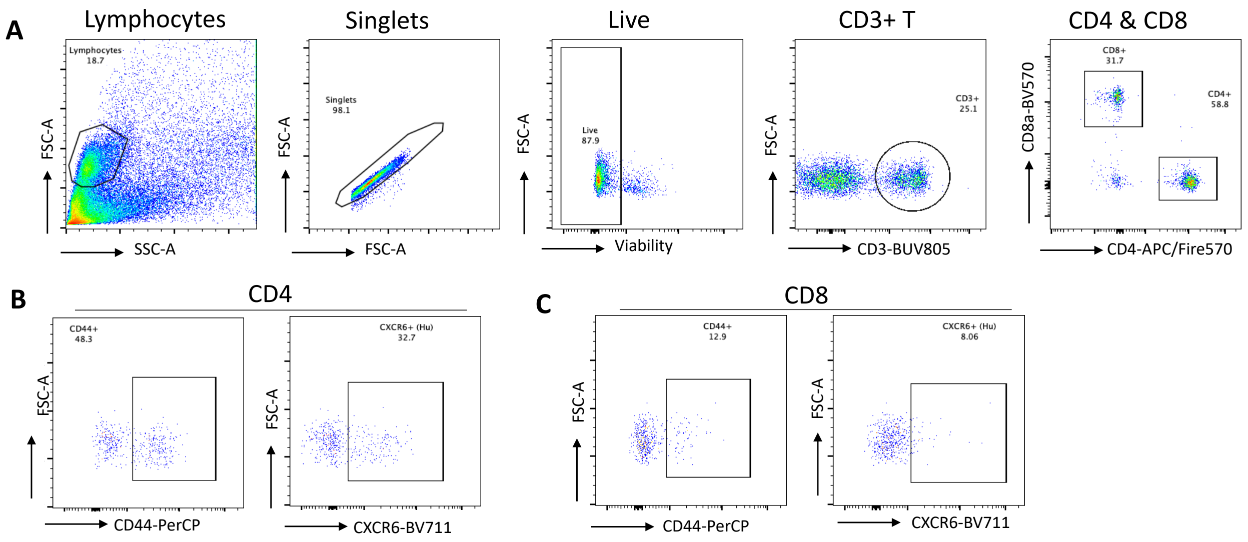

Supplementary Figure 4. Gating strategy for T cell analysis in the BAL. Mice were vaccinated intramuscularly with vaccines on a prime-boost schedule at week 0 and 3. Two weeks after booster dose (week 5), mouse BAL was harvested and subjected to immune staining and flow cytometry. **(A)** Lymphocytes were gated based on FSC-A and SSC-A, followed by gating on singlets. Fixable viability dye was used to differentiate live and dead cells, followed by gating on live CD3+ T cells. T cells were further gated for CD4 and CD8 T cell subsets. Next, CD44 as a marker of T cell activation, and CXCR6 as a marker of cells with tissue residency potential, were gated in CD4 **(B)** and CD8 **(C)** T cells.

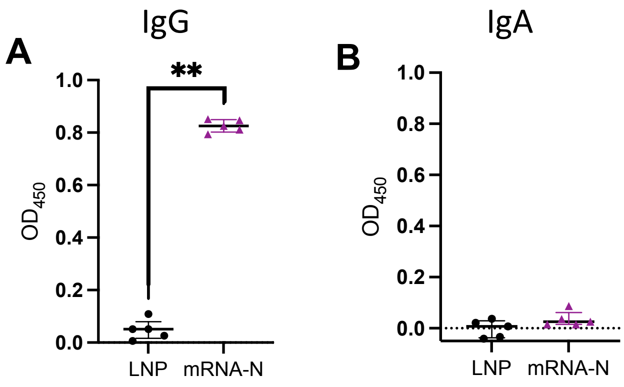

Supplementary Figure 5. Nucleocapsid specific antibody response in the BAL. ELISA was performed to examine N binding IgG and IgA in BAL samples. All samples underwent 1:1 dilution for ELISA. **(A-B)** ELISA measurement of N-specific binding **(A)** IgG and **(B)** IgA after vaccination (week 5). OD_{450} values for individual samples after 1:1 dilution are shown. Data are presented as medians +/- IQR. Mann-Whitney test was used for statistical analysis. **$p<0.01$

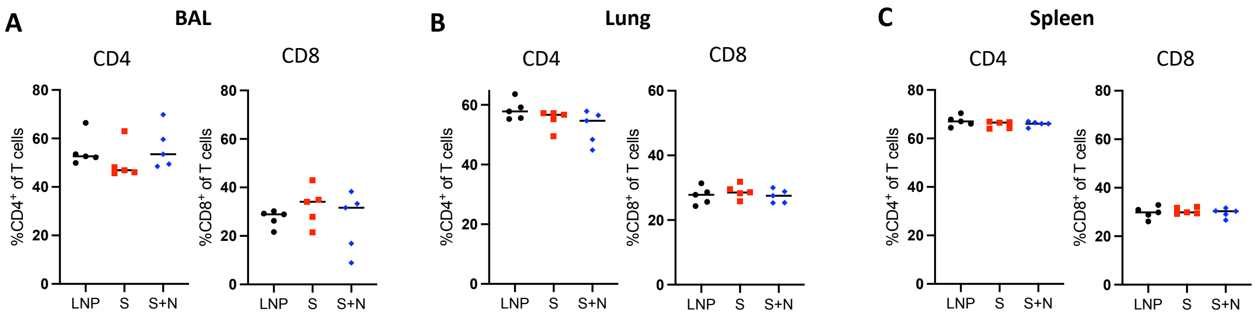

Supplementary Figure 6. Total T cells in the lung, BAL, and spleen after empty LNP, mRNA-S, or mRNA-S+N vaccination. Mice were vaccinated intramuscularly with vaccines on a prime-boost schedule at week 0 and 3. Two weeks after booster dose (week 5), lungs and BAL samples were harvested and subjected to staining and flow cytometry. Percentages of CD4 and CD8 T cells in the lungs **(A)**, in the BAL **(B)**, and in the spleens **(C)** were measured and compared among the three groups (LNP as mock, mRNA-S, and mRNA-S+N). Horizontal lines indicate mean.

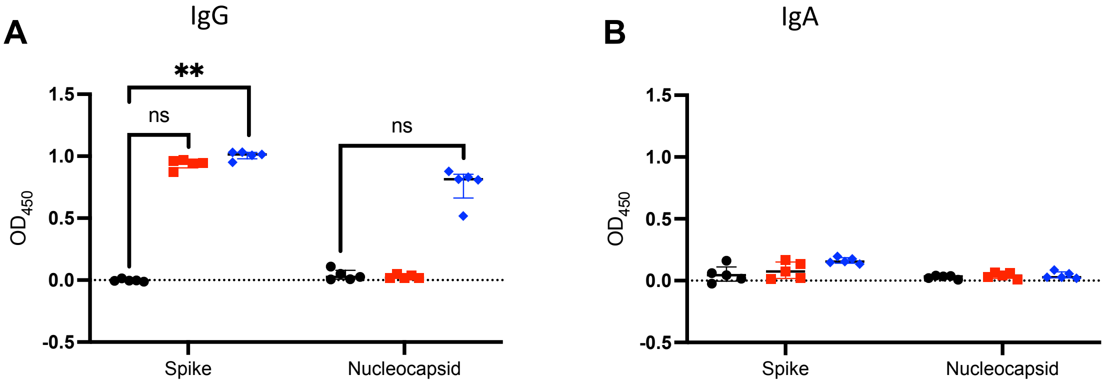

Supplementary Figure 7. S- and N-specific binding antibodies in the BAL. ELISA was performed to examine antigen-specific binding antibodies in BAL samples. All samples were diluted 1:1 prior to ELISA. **(A-B)** ELISA measurement of S- and N-specific binding IgG (A) or IgA (B) after vaccination (week 5). OD_{450} values for individual samples are shown. Data are presented as median. Kruskal-Wallis test was used for statistical analysis. **p<0.01
